# Exploring community knowledge, perceptions, and the impacts of anthrax among farming communities living in game management areas in Zambia: A qualitative study using a hybrid approach

**DOI:** 10.1371/journal.pntd.0012852

**Published:** 2025-05-08

**Authors:** Chisoni Mumba, Davies Phiri, Exillia Kabbudula, Laila Gondwe, Noanga Mebelo, Gubula Simweene, Mutinta N. Hankolwe, Kezzy Besa, Suwilanji S. Sichone, Mwila Kayula, Mainda Geoffrey, Kivaria M. Fredrick, Charles Bebay, Soumare Baba, Mtui-Malamsha N. Jesse, Suze P. Filippini, Chitwambi Makungu

**Affiliations:** 1 Department of Disease Control, School of Veterinary Medicine, University of Zambia, Lusaka, Zambia; 2 Department of Community Education and Lifelong Learning, University of Zambia, Lusaka, Zambia; 3 Department of Aquaculture and Fisheries Sciences, Lilongwe University of Agriculture and Natural Resources, Lilongwe, Malawi; 4 Pangolin Protection, Wild Crime Prevention (WCP), Chilanga, Zambia; 5 Emergency Centre for Transboundary Animal Diseases (ECTAD). The Food and Agriculture Organization of the United Nations (FAO), Lusaka, Zambia; 6 Emergency Centre for Transboundary Animal Diseases (ECTAD). The Food and Agriculture Organization of the United Nations (FAO), Nairobi, Kenya; 7 Emergency Centre for Transboundary Animal Diseases (ECTAD). The Food and Agriculture Organization of the United Nations (FAO), Rome, Italy; Oklahoma State University, UNITED STATES OF AMERICA

## Abstract

Anthrax remains a neglected zoonotic disease of critical public and animal health significance in Zambia, particularly in regions with active human-wildlife-livestock interfaces such as the Western, Southern and Eastern provinces of Zambia. This study explores the socio-ecological drivers of anthrax transmission and examines the role of legal and illegal wildlife trade value chains in sustaining outbreaks. Secondly, the study explores the methodology used to investigate community knowledge, perceptions, and the impacts of anthrax through focus group discussions (FGDs) and a hybrid approach combining traditional thematic analysis with artificial intelligence (AI) tools. The research was framed within the interpretivist paradigm, aiming to understand shared experiences and socio-cultural contexts. The study utilized focus groups to encourage interaction and generate rich, collective insights. The hybrid approach allowed for data analysis that combined researcher-led reflexivity with AI-driven thematic analysis.

Findings reveal diverse levels of awareness about anthrax, widespread misconceptions, and the influence of cultural beliefs on health behaviours. Communities linked anthrax outbreaks to interactions with wildlife and the illegal game meat trade, highlighting the complex interplay of ecological, economic, and behavioural factors in disease dynamics. Additionally, the study underscores the socioeconomic toll of anthrax, including livestock losses, disrupted livelihoods, and food insecurity, compounded by inadequate public health and veterinary responses.

The insights gained from this research emphasize the need for multi-sectoral interventions tailored to the specific needs of these communities.

## Author summary

Anthrax is a zoonotic disease of significant public health, veterinary, and ecological concern in Zambia, particularly in regions where human-wildlife-livestock interactions are frequent. This study focuses on understanding the socio-ecological factors driving anthrax transmission in communities near Game Management Areas. Using a combination of focus group discussions and an innovative hybrid data analysis approach that blends traditional qualitative methods with artificial intelligence tools, the research explores community knowledge, perceptions, and the impacts of anthrax. Key findings reveal diverse levels of awareness about anthrax, widespread misconceptions, and the influence of cultural beliefs on health behaviours. Communities often link anthrax outbreaks to interactions with wildlife and the illegal game meat trade, highlighting the complex interplay of ecological, economic, and behavioural factors in disease dynamics. Additionally, the study underscores the socioeconomic toll of anthrax, including livestock losses, disrupted livelihoods, and food insecurity, compounded by inadequate public health and veterinary responses. The insights gained from this research emphasize the need for multi-sectoral interventions tailored to the specific needs of these communities. These include strengthening disease surveillance, promoting culturally sensitive education and awareness campaigns, improving veterinary services, and addressing systemic drivers such as poverty and food insecurity. Adopting a One Health approach, this study provides actionable recommendations to mitigate anthrax risks and enhance resilience in affected communities, contributing to sustainable public and animal health outcomes.

## 1. Introduction

Anthrax is a neglected zoonotic disease that is a significant public and animal health concern in Zambia [1,2]. The disease is caused by *Bacillus anthracis*, a resilient pathogen capable of surviving in harsh environmental conditions for extended periods through spore formation. Globally, anthrax remains a significant challenge in areas with favourable environmental conditions, such as alkaline soils and arid or semi-arid climates. In Zambia, anthrax is endemic in the Western and Eastern provinces, where human, livestock, and wildlife interactions are deeply intertwined [2,3].

Anthrax outbreaks in Zambia have been recorded in humans, livestock, and wildlife species, with significant socioeconomic and ecological impacts [4]. Historical evidence highlights outbreaks involving diverse wildlife species, including kudus, hippos, giraffes, buffaloes, and domestic cattle [5]. These outbreaks have also resulted in human fatalities, primarily linked to the consumption of infected meat or exposure to infected animals. For example, the gastrointestinal form of anthrax was reported in humans during outbreaks in the 1990s, with over 200 cases recorded in 1990 alone, resulting in a high case fatality rate of 19.1% [3,6]. More recently, anthrax outbreaks in 2023 in the Lumezi District of the Eastern province of Zambia were associated with hippo mortalities, underscoring the continued public health risk and the challenges of managing the disease in multi-host systems [7–9].

The livestock-human-wildlife interface plays a critical role in the transmission and maintenance of anthrax in endemic regions. Western and Eastern provinces of Zambia, characterized by vast national parks and communal grazing areas, are hotspots for anthrax outbreaks due to frequent interactions between humans, livestock, and wildlife [1,10,11]. Wildlife species, particularly herbivores such as antelopes and hippos, act as reservoirs for the disease, perpetuating the infection cycle in livestock and humans [12–14]. The ecology of *Bacillus anthracis* in these regions is influenced by environmental factors such as soil alkalinity and climatic conditions, which favour the persistence of spores in the environment [5].

Illegal and legal value chains in wildlife trade further complicate disease management in these regions. Illegal activities, such as poaching and bushmeat trade, often coexist with legitimate wildlife-based economies and provide avenues for the spread of zoonotic diseases like anthrax [4]. Studies suggest that these illegal value chains are highly resilient and thrive even in the presence of formal market regulations [15,16]. In Zambia, the socioeconomic dynamics of communities living near national parks and limited enforcement and veterinary services exacerbate the challenges of controlling anthrax outbreaks [1,11].

The significance of livestock-human-wildlife interface areas cannot be overstated [17]. These regions are crucial for the livelihoods of local communities, who depend on livestock for food, income, and cultural practices. However, the interface also presents a heightened risk for zoonotic disease transmission [1]. Livestock can become infected through grazing on contaminated pastures, leading to spillover events in humans who handle or consume infected meat. The absence of robust veterinary infrastructure, early detection systems, and public awareness further increases the vulnerability of these communities to anthrax outbreaks [11,18].

Despite the clear risks, significant gaps remain in understanding the complex interplay of ecological, socioeconomic, and behavioural factors that drive anthrax transmission in Zambia. Existing literature highlights the role of socio-demographics, moral and social norms, and the legitimacy of governance structures in shaping human behaviours related to disease prevention and control [1,11,19,20]. However, the determinants of anthrax transmission within wildlife trade value chains, both legal and illegal, remain underexplored.

In this study, we address these knowledge gaps by examining the characteristics of wildlife trade value chains and investigating the socio-ecological determinants of anthrax transmission in communities near national parks. We focused on areas at the livestock-human-wildlife interface to provide a deeper understanding of the factors influencing the distribution and persistence of anthrax in Zambia and inform strategies for effective disease control and prevention.

## 2. Methodology

### Ethics statement

We obtained ethical clearance from Excellence in Research Ethics and Science (ERES) Converge, reference number “2024-Aug-015.” Given the sensitivity of the topic—the illegal trade in game meat—ensuring participant confidentiality was critical to protecting individuals from potential legal repercussions or social stigmatization. To this end, no names of individuals were mentioned, and no pictures were shared to maintain anonymity.

We anonymized names of participants in the raw narratives using pseudonyms (A1, A2, A…) to further safeguard confidentiality. We also de-identified all participant responses during transcription and securely stored raw data in compliance with ethical guidelines. We fully informed participants about the study’s objectives and assured them that their participation was voluntary, with the option to withdraw without any negative consequences. Formal verbal consent was obtained from participants.

### 2.1. Study design

We employed a qualitative research design, combining traditional thematic analysis with the use of artificial intelligence (AI) tools. This hybrid approach ensured both the depth provided by human-led analysis and the efficiency and precision afforded by AI technologies, reflecting a commitment to robust qualitative research that combines reflexivity with innovation [21,22].

### 2.2. Description of the study sites

**Fig 1:**
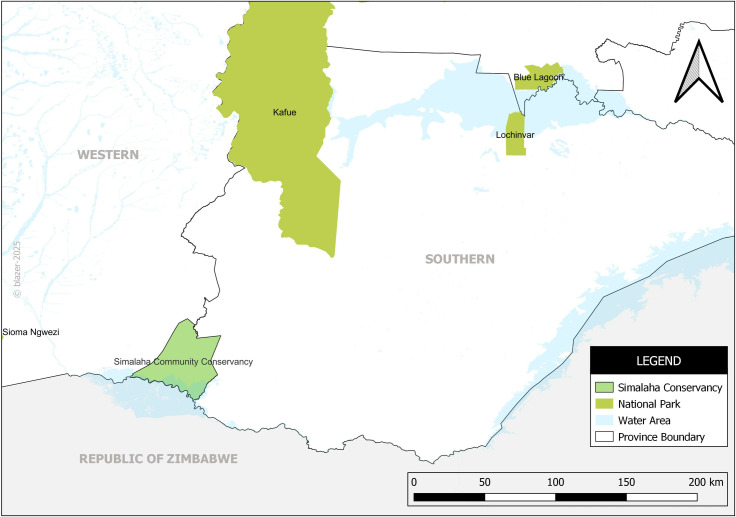
Map showing the study sites. Source: Developed by authors. Reprinted from [Zambia_Mosaic_250Karc1950_ddecw] under a CC BY licence, with permission from the Surveyor General, Government Republic of Zambia, original copyright, [1974].

We conducted the study in Simalaha Community Conservancy, Lochinvar, and Blue Lagoon National Parks, as shown in Fig 1. Simalaha Conservancy is located in Zambia’s Western Province. This conservancy encompasses communities from Mwandi and parts of Sesheke districts. Simalaha plays a critical role in wildlife conservation and community livelihoods, as it lies within one of the six key wildlife dispersal corridors of the Kavango-Zambezi Transfrontier Conservation Area (KAZA TFCA) [23]. Specifically, it is part of the Chobe-Zambezi dispersal area, which connects Chobe National Park in Botswana with Kafue National Park in Zambia. This corridor facilitates the migration and movement of key wildlife species, contributing to biodiversity conservation in the region.

The Simalaha Conservancy is also notable for its community-driven approach to conservation, where local people actively manage natural resources and promote sustainable coexistence between humans and wildlife [23]. However, this proximity to wildlife brings challenges, including an increased risk of zoonotic disease transmission, such as anthrax, which thrives in areas of close human-wildlife-livestock interactions. The ecological and socioeconomic importance of this region makes it an ideal site for studying the complex dynamics of anthrax transmission and identifying interventions that can balance public health and conservation goals.

The other study sites are Lochinvar and Blue Lagoon National Parks, located within the Kafue Flats in Zambia (Fig 1). We specifically selected these study sites due to the endemicity of anthrax in areas surrounding most national parks, highlighting their importance in understanding disease dynamics at the human-wildlife-livestock interface.

Lochinvar National Park, situated on the southern edge of the Kafue Flats, encompasses a vast floodplain formed by the Kafue River between the Itezhi-Tezhi dam to the west and the Kafue Gorge to the east. The park is renowned for its rich biodiversity, particularly its significant populations of Kafue lechwe, a semi-aquatic antelope species endemic to Zambia and a wide variety of bird species. However, its ecological significance also brings challenges, such as the heightened risk of zoonotic diseases like anthrax, which thrives in floodplain environments characterized by seasonal water fluctuations and nutrient-rich soils [10].

Blue Lagoon National Park, located approximately 120 kilometres west of Lusaka on the Kafue Flats, shares similar ecological and conservation importance. It serves as a critical habitat for wildlife, including waterbirds and other herbivore species, while also supporting local communities through tourism and ecosystem services [24]. The proximity of both parks to human settlements increases the likelihood of interactions between wildlife, livestock, and humans, thereby amplifying the potential for anthrax outbreaks.

### 2.3. Theoretical framework of the study

We framed the study within the interpretivist paradigm to allow us to understand the shared experiences and socio-cultural contexts of study participants in relation to anthrax within their communities. We used Focus Group Discussions (FGDs) as the primary data collection method because they encourage interaction between the study participants and generate rich, collective insights that aim to capture diverse perspectives on anthrax-related knowledge, practices, and challenges. For data analysis, we combined researcher-led reflexive thematic analysis with an AI tool to allow us to identify themes while exploring relationships within the data. This hybrid approach fits into the increasing recognition of methodological pluralism in qualitative research [21,22].

### 2.4. Data collection

#### 2.4.1. Participants and sampling.

We used gatekeepers (community leaders) to purposively select participants from communities in regions affected by anthrax outbreaks and ensured diversity in gender, age, occupation, and geographical location. The selection process emphasized individuals whose livelihoods, beliefs, or practices intersected with anthrax risk. These included livestock farmers, game meat traders, and community leaders. In the end, we conducted Six FGDs, each comprising 8–12 female and male participants only, to facilitate a comprehensive exploration of community-specific experiences. Table 1 shows the location, number of FGDs conducted, number of participants per group and language used.

**Table 1:** Information on FGDs in study locations.

Location	Number of FGDS	No. of female participants	No. of male participants	Language used
Monze	2	8	8	Tonga
Simalaha	2	9	12	Lozi
Mumbwa	2	8	10	Ila

#### 2.4.2. *Procedure of focus group discussions.*

We developed a checklist FGD guide to maintain consistency across discussions and allow flexibility. The guide contained open-ended questions that allowed participants to share their knowledge of anthrax, including symptoms, transmission, and prevention; the influence of cultural beliefs and practices on perceptions of anthrax; the economic and social impacts of anthrax outbreaks on livelihoods and community dynamics; and the perceived role of wildlife as potential sources of anthrax. Each FGD was a minimum of 60 minutes to a maximum of 90 minutes. The discussions were in the daily language of each participating community (Silozi, Ila, and Tonga). Audio recordings were transcribed verbatim and translated into English by independent Master of Science students (authors) who were native speakers of the local languages for analysis.

### 2.5. Data analysis

We employed a hybrid approach combining researcher-led manual and AI-assisted analysis to ensure a comprehensive qualitative data analysis. This allowed us to leverage the strengths of different analytical tools while mitigating potential researcher biases.

#### 2.5.1. Reflexive thematic analysis.

This stage involved a researcher-led thematic analysis informed by Braun and Clarke [25] framework. We immersed ourselves in the data through repeated readings of the transcripts, identifying initial patterns, and generating codes to capture meaningful segments. We then recoded codes and grouped them into preliminary themes, which were peer-reviewed, refined, and defined to ensure coherence, distinctiveness, and relevance to the research questions. Finally, we synthesized themes into cohesive narratives supported by direct quotes from participants. For instance, under the theme “Community Knowledge and Perceptions of Anthrax,” the analysis revealed misconceptions about anthrax, with one participant stating, “People do not believe that eating game meat can lead to anthrax because they have been eating it for a long time” (Monze Men, A1). This insight provided a foundation for exploring how traditional beliefs influence health behaviors [21].

#### 2.5.2. AI-assisted analysis.

***Gemini 1.5:*** First, we used Gemini, a Large Language Model (LLM) developed by Google AI, to help us identify patterns and cluster-related narratives within the data. Its ability to process huge amounts of textual data helped map connections between concepts and identify recurring themes across the FGD transcripts. For example, Gemini unearthed a strong connection between discussions of the illegal game meat trade and concerns about anthrax outbreaks. It revealed consistent mentions of specific trade routes, concealment methods, and anxieties surrounding the risks associated with consuming illegally obtained meat by the participating communities. These insights contributed to the development of the theme “Illegal Game Meat Trade and Anthrax Risk,” providing a comprehensive overview of community perspectives on this critical aspect of anthrax transmission [26].

***ChatGPT 4.0:*** Second, we used ChatGPT, another LLM developed by OpenAI, for interpretations of data by exploring relationships between themes and underlying contexts as guided by Morgan [27]. ChatGPT has the ability to engage in contextualized reasoning, which allowed us to uncover deeper connections between participants’ statements and their cultural beliefs, socioeconomic circumstances, and lived experiences. For instance, ChatGPT highlighted how traditional beliefs and religious narratives shaped perceptions of anthrax and influenced community responses to control measures. It identified instances where participants drew upon religious texts or traditional knowledge to explain the causes of anthrax and justify their practices related to meat consumption and animal health. These insights enriched the theme “Community Knowledge and Perceptions of Anthrax” as it provided a deeper understanding of the cultural and social factors that shape community responses to the disease [22].

**AILYZE 2.3:** Third, we employed AILYZE, a qualitative data analysis software that uses Natural Language Processing (NLP) and Machine Learning (ML) techniques to conduct sentiment analysis on the FGD transcripts. This allowed us to capture the emotional tone and subjective experiences expressed by participants in their narratives. AILYZE identified instances where participants’ narratives exhibited anxiety and fear, particularly in discussions about the risks of consuming infected meat and the economic losses caused by livestock deaths. It also detected expressions of frustration and mistrust towards formal health campaigns and authorities by highlighting the emotional impact of anthrax outbreaks on communities. These insights added depth to the theme “Impact of Anthrax on Communities” by providing an understanding of the emotional landscape surrounding the disease and its consequences [26].

#### 2.5.3. Thematic synthesis and validation.

**Manual integration across domains**: Following AI-assisted thematic identification, we manually integrated themes across the community knowledge, perceptions, and the impacts of anthrax domains. The AI-generated outputs served as a guide, but the final synthesis relied on human judgment to ensure contextual accuracy. The integration process involved comparing AI-generated themes with direct quotes from the transcripts to maintain authenticity and validity.

**Validation of themes**: We conducted multiple rounds of validation to enhance credibility. We cross-referenced each theme with verbatim quotations to ensure it accurately represented the respondents’ perspectives. Themes were also reviewed independently by multiple researchers during a data analysis workshop to eliminate potential biases and inconsistencies. These refinements ensured that the final themes were precise and accurately reflected the data.

**Final theme development**: We developed the final themes iteratively, incorporating AI insights while grounding them in qualitative evidence. This phase involved a systematic review of all coded data, ensuring that each theme was backed by multiple instances across the dataset. We structured the themes to align with the core areas of the study providing a well-rounded analysis.

#### 2.5.4. Integrative synthesis.

The final analysis phase synthesized insights from both human-led and AI-supported approaches to ensure coherence and depth in the findings. AI-generated results were critically analyzed and used to complement rather than replace human interpretations. We cross-referenced AI findings with participant narratives and contextualized realities to ensure that the emergent themes were both robust and grounded in the lived experiences of participants [21,22,27].

For example, Gemini 1.5 flagged the illegal game meat trade as a significant theme by clustering mentions of trade routes and practices. However, these findings alone did not explain the underlying motivations. The research team contextualized them within socioeconomic drivers such as poverty, food insecurity, and limited access to legal meat sources, enriching the theme by linking risky behaviors to broader systemic challenges.

ChatGPT 4.0 added interpretive nuance, uncovering cultural narratives such as perceptions of anthrax as divine punishment or its attribution to traditional beliefs. These insights prompted deeper exploration, with the research team cross-referencing AI outputs against participant statements to ensure accuracy and alignment with cultural contexts. This enabled a richer understanding of themes such as “Beliefs and Cultural Practices.”

AILYZE 2.3 contributed an emotional dimension by detecting heightened anxiety and mistrust in discussions about meat consumption during outbreaks. This sentiment analysis complemented manual findings, where researchers linked fear to past outbreaks and distrust in informal meat markets. Incorporating emotional tones deepened the understanding of themes like “Social Impacts” and “Risk Perceptions.”

We integrated the strengths of both approaches to produce final themes that were analytically rigorous and reflected participant voices. This synthesis demonstrated AI’s potential as an analytical aid while highlighting the essential role of human expertise in capturing complexity and nuance.

### 2.8. Reporting of findings

The findings were presented in a descriptive narrative format, supplemented by verbatim quotations to illustrate key themes. Each conclusion drawn was grounded in direct evidence from the transcripts, ensuring transparency and allowing for independent verification. The reporting prioritized clarity and coherence, making the results accessible to academic and non-academic audiences.

## 3. Results and discussion

### 3.1. Community knowledge and perceptions of anthrax

This theme explores the understanding and awareness of anthrax across different communities. The analysis revealed varying levels of knowledge, misconceptions, and traditional beliefs that influence perceptions of the disease and its transmission.

#### 3.1.1. Awareness and knowledge of anthrax.

Some communities demonstrated familiarity with anthrax, recognizing its symptoms in humans and animals. This familiarity was often linked to prior outbreaks or sensitization campaigns.

***Mumbwa women*** *: A5: “Anthrax is a very dangerous and deadly disease. It kills within a short period of time.”*

***Monze men*** *: A9: “Anthrax is a very contagious disease. It spreads to anything that comes into contact with an infected animal; it’s a deadly disease. It kills within a short period of time and it also causes sores.”*

However, other communities exhibited limited knowledge, with some members unable to identify anthrax symptoms or understand its transmission.

***Mumbwa men*** *: A8: “This disease, we do not know about it, not even the sign. We are only beginning to hear about it now through phones, radio, etc. We have never seen meat from a diseased animal; if we are shown that this is how the meat looks, we can know. But at the moment, we know nothing.”*

This contrast in awareness and knowledge suggests the need for targeted educational interventions to ensure that all community members have a basic understanding of anthrax and its risks. Men seemed more aware of the **transmission pathway** of anthrax, often associating it with wildlife and livestock, while women focused more on the **severity and consequences** of the disease.

#### 3.1.2. Understanding of transmission.

The communities generally understood that anthrax could be transmitted from animals to humans through the consumption of infected meat.

***Monze men*** *: A4: “Anthrax is found in animals, specifically cattle, goats, and wild animals. If you come into contact with an infected animal without knowing that it is infected, you also get infected.”*

***Mumbwa men*** *: A7: “All animals dying from anthrax must be handled properly and buried or burnt if you cannot bury them because anthrax is a very contagious disease and can spread quickly through the air.”*

The awareness of animal-to-human transmission highlights the importance of safe meat handling practices and proper disposal of infected carcasses. However, the presence of misconceptions about transmission underscores the need for educational programs to address these misunderstandings and provide clear information about how anthrax is spread. This is in agreement with Sitali [11]

#### 3.1.3. Beliefs and practices.

Cultural beliefs and traditional practices shaped community perceptions and behaviors related to anthrax. Some communities readily accepted the link between meat consumption and anthrax, while others attributed the disease to witchcraft or other beliefs.

***Monze men*** *: A1: “People do not believe that eating game meat can lead to anthrax because they have been eating it for a long time. Even biblically, it says there would be diseases and that is why certain foods should not be eaten because of diseases. But after the floods, God allowed eating meat. So others believe this while others do not.”*

The influence of cultural beliefs and traditional practices on perceptions of anthrax underscores the need for culturally sensitive interventions that respect local traditions while promoting safe behaviors. Studies for anthrax and other contagious diseases in domestic animals in Western province highlighted similar findings [1,11,28]. Men seemed to **reject the connection** between anthrax and meat consumption, believing they have always eaten it safely, while women acknowledged **traditional and religious narratives**, but also express **greater concern for disease prevention**.

#### 3.1.4. Perceptions of risk.

Communities perceived the risk of anthrax differently depending on the source. Some viewed wildlife as the primary source, while others considered livestock or meat from unknown origins as risk factors.

***Simalaha men*** *: A8: “Anthrax is a dangerous, deadly disease involving all animals especially buffaloes, elephants, lechwes, cattle and goats. It also affects livestock and spreads from wild animals to livestock.”*

***Monze women****: A2: “This disease comes as a result of consuming meat especially uncertified meat that is of unknown origin*.”

These varying perceptions highlight the need to understand community-specific views when designing interventions. Educational programs and risk communication strategies should be tailored to address the specific concerns and beliefs of each community, emphasizing the potential risks associated with different sources of anthrax [20].

### 3.2. Impact of anthrax on communities

This theme delves into the consequences of anthrax outbreaks on the social and economic well-being of communities. The analysis highlights the disruption of livelihoods, emotional distress, and social tensions associated with the disease.

#### 3.2.1. Economic impacts.

The economic impacts of anthrax outbreaks were evident in the data, with participants expressing concerns over livestock deaths and the financial burden of disease control.

***Simalaha women*** *: A4: “That time when the disease was at its peak, I lost 14 adult cattle, and I cannot even count how many calves I lost. The calves cannot survive once the cows die.”*

The loss of livestock affects food security and disrupts income generation and livelihoods, particularly in communities where livestock plays a crucial role in the local economy [17]. Men emphasize **loss of income** due to cattle deaths, while women highlight the **impact on household food security** and stress the burden of caring for sick family members.

#### 3.2.2. Social impacts.

The social impacts of anthrax outbreaks were equally significant. Participants described fear, anxiety, and mistrust within communities during outbreaks.

***Monze women*** *: A2: “The disease brought fear among the community members especially around meat eating habits. People used to buy meat from one another in the past but from the time the disease broke out, the trend has changed among many though a few still eat meat.”*

***Mumbwa men*** *: A9: “Even if we are saying we bury the animals, it is just from our knowledge of the disease that we say that. Otherwise, we have never experienced anthrax here. What we do here, whereby we take the dried meat to Lusaka, even those in Southern Province do it.”*

The disease could lead to social tensions and conflicts, particularly when suspicions of illegal meat sales or poaching arise. The emotional distress caused by the disease and its potential impact on relationships and community cohesion were also evident in the data. The practice of drying the meat from anthrax-contaminated carcasses and selling it in other town under the disguise of dry game meat explains why Zambia has reported human anthrax cases away from outbreak hotspots [7,29].

#### 3.2.3. Health impacts.

The health impacts of anthrax were evident in the descriptions of human cases, highlighting the physical suffering and potential mortality associated with the disease.

***Simalaha men*** *: A1: “What we understand about anthrax is that it is a disease which kills. It is not just mere understanding, but we have experienced it. We have seen it, and we have seen the symptoms.”*

***Simalaha women*** *: A4: “This same anthrax, last year some young lad got anthrax, she lives in Simalaha. She was taken to the hospital and was treated and briefly recovered, but as we speak today, she passed away.”*

These accounts underscore the serious health consequences of anthrax infection, including the potential for long-term suffering and even death. The fear of contracting the disease and the challenges in accessing healthcare were also evident in the data.

### 3.3. Role of wildlife in anthrax transmission

This theme further dissects the intricate relationship between wildlife and anthrax transmission. The analysis reveals the complex interplay of factors, including wildlife as a source of infection, cross-border animal movement, and the impact of human-wildlife interactions.

#### 3.3.1. Wildlife as a source of infection.

The data highlights the role of wildlife, particularly buffaloes, as a significant reservoir and source of anthrax infection for livestock and humans. The proximity of wildlife habitats to human settlements and grazing areas creates opportunities for interspecies interaction and increases the risk of transmission.

***Simalaha men*** *: A7: “This disease called anthrax, my father said it started from Botswana. The disease came from the buffaloes from Botswana and that is how it ended up here. The buffaloes got very sick and started dying. Eventually, last year, we experienced this disease and actually started knowing about it.”*

***Simalaha women*** *: A4: “The buffaloes were dying in numbers, but we were not told what was actually killing them. That information was purposely kept away from us. Then we noticed that our cattle started dying rapidly, and that is when there was panic, and we were told that it was anthrax killing them and we should quickly be burying the animals that die.”*

These excerpts demonstrate the perceived link between wildlife (buffaloes) and the introduction and spread of anthrax within communities. The presence of infected wildlife in shared grazing areas poses a direct threat to livestock, which can then transmit the disease to humans through consumption or contact.

#### 3.3.2. Cross-border movement of wildlife.

Wildlife movement across international borders adds another layer of complexity to anthrax transmission. Participants in the Simalaha FGDs, in particular, highlighted the role of transboundary wildlife movement in the introduction and spread of anthrax.

***Simalaha women*** *: A2: “When I was younger, in 2003, this disease once occurred at the river. The cattle died in large numbers. The people from the veterinary offices then came and gave the animals some medication, and the deaths stopped. So, from that time, we have not experienced this disease, and our animals really multiplied until two years ago when they brought the wildlife into the park, and the animals started dying.”*

This cross-border movement of wildlife makes disease surveillance and control more challenging, requiring collaborative efforts between neighboring countries to manage the spread of anthrax effectively.

#### 3.3.3. Human-wildlife interactions.

Human-wildlife interactions, such as hunting, farming in wildlife areas, and contact with wildlife carcasses, can significantly influence anthrax transmission dynamics.

**Simalaha women**: A2: “So, now the problem is that even the hippos are bringing anthrax here. The hippos graze in the same places as our animals and you find that all our animals (cattle, goats and pigs alike) are now getting sick because the hippos had anthrax and the ones dying were being burnt.”

These quotes illustrate how human activities, including contact with potentially infected carcasses and sharing grazing areas, can increase the risk of anthrax transmission [11]. Understanding these interactions is crucial for developing targeted interventions that minimize the risk of human and livestock exposure to the disease.

### 3.4. Illegal game meat trade and anthrax risk

This theme examines the prevalence, methods, and market dynamics of the illegal game meat trade, emphasizing the heightened risk of anthrax transmission associated with this illicit activity.

#### 3.4.1. Prevalence of illegal trade.

Despite regulations and restrictions, the data underscores the pervasiveness of the illegal game meat trade. This illicit trade is often driven by economic factors, cultural preferences for game meat, and its perceived high value.

***Mumbwa men*** *: A9: “When we used to poach, we used to eat a lot of game meat. It was abundant, but now it is very difficult to find. Now, for you to eat game meat, you have to get it from the game rangers after they have stolen it, and it is just by chance”.*

***Mumbwa women*** *: A6: “Some months ago, game licences were easy to purchase, but poachers were also rampant; they would kill animals illegally without licences”.*

Despite legal restrictions, the persistence of this trade indicates the presence of complex factors driving the demand and supply of illegal game meat. These factors may include economic incentives, cultural preferences, and limited access to legal sources of game meat.

#### 3.4.2. Transportation and concealment.

The illegal game meat trade involves sophisticated networks and methods to avoid detection and circumvent regulations. These include using informal transportation routes, concealing meat in other goods, and, in some cases, bribing officials.

**Mumbwa men**: A9: Yes, we carry it on the bicycle. We dry it first by roasting the meat. You kill the animal first, and then while it is still fresh, you put it over a fire for the blood and the water to drain out. Then, you make bundles that fit into sacks, apply perfumes to sacks, and smuggle.

**Monze men**: A7: “We consult what is happening on the road before we start off; there are some communications that go on. We just do not want to tell you what goes on in our business. Meaning there is a chain of informers on the way? We just do not want to tell you”.

The use of covert transportation routes, concealment techniques, and communication networks demonstrates the organized nature of this trade. This organized nature highlights the challenges faced by authorities in regulating and controlling the illegal game meat trade, which is a high-risk factor for anthrax transmission and spread.

### 3.5. Market dynamics

Factors such as demand, supply, and pricing influence the market dynamics of the illegal game meat trade. The high demand for game meat and restrictions on legal access create a lucrative market for illegal traders.

***Mumbwa women*** *: A3: “The illegal one is cheaper. It is only bad when you are caught. If you are not caught, it is good.”*

***Simalaha women*** *: A4: “The fast business I was talking about is a situation whereby there are a lot of canters (light trucks) moving from Livingstone and going to the river from Monday to Saturday. So, once you kill an animal, you can just load it.”*

These quotes suggest that the illegal game meat trade is driven by economic incentives, with price playing a significant role in consumer choices. The availability of cheaper, albeit illegally obtained game meat can attract consumers despite the associated risks.

### 3.6. Risk of anthrax from illegal game meat

The consumption of illegally obtained game meat poses a heightened risk of anthrax transmission. This is because meat from illegal sources often bypasses veterinary inspection and certification processes, increasing the likelihood of infected meat entering the market.

***Monze women*** *: A5: The cases we saw were on people that are well known for eating cattle that die on their own, so we have great suspicion that the cases were related to uncertified beef than game meat.*

***Simalaha men*** *: A5: “Another thing I have noticed here is that the people who take fish to the Kasumbalesa border point are also a problem. You will find that they stay here and do their fishing until the time to go draws near. Once this happens, they either get animals that have died on their own or hunt down animals and kill them, properly service them, and put them in the middle of the fish in disguise”.*

These excerpts highlight the elevated risk of anthrax associated with illegal game meat consumption. The lack of regulation and oversight in the illegal trade increases the chances of consumers being exposed to infected meat, posing a significant public health concern.

### 3.7. Community engagement and interventions

This theme emphasizes the importance of community involvement in addressing anthrax. It highlights the need for collaborative and sustainable interventions that incorporate local knowledge and address the root causes of the disease.

#### 3.7.1. Need for engagement.

The data consistently points to the necessity of actively engaging communities in anthrax prevention and control efforts. Participants emphasized the importance of collaboration between community members, traditional leaders, healthcare providers, and veterinary services.

***Monze women*** *: A1: We need to engage the chiefs and the indunas so that they can help us with sensitization.*

***Simalaha men*** *: A3: We need education and sensitization on this disease, and people need to be taught how to prevent it.*

Community engagement is crucial for ensuring that interventions are contextually appropriate, culturally sensitive, and address the specific needs and concerns of the affected populations [20].

#### 3.7.2 .Sustainable interventions.

Participants emphasized the need for interventions that address the underlying causes of anthrax transmission, such as poverty, food insecurity, and the illegal wildlife trade. They highlighted the importance of long-term solutions that promote sustainable livelihoods and improve community resilience.

***Simalaha Men*** *: A6: “We need to find ways to improve our livelihoods so that we are not forced to rely on illegal activities like poaching”.*

***Mumbwa Women*** *: A4: “If people had other sources of income, they would not be forced to sell meat from animals that have died from anthrax”.*

Sustainable interventions should go beyond immediate disease control measures and address the socioeconomic factors that contribute to anthrax risk. This may involve promoting alternative livelihoods, improving access to education and healthcare, and strengthening community-based natural resource management.

#### 3.7.3. Education and awareness.

The data consistently highlights the critical role of education and awareness campaigns in empowering communities to protect themselves from anthrax. Participants emphasized the need for clear, accurate, and accessible information about the disease, its transmission, and prevention measures.

***Mumbwa men*** *: A5: “People need to be educated on the dangers of eating meat from animals that have died from anthrax.”*

***Monze women*** *: A3: “We need more awareness campaigns to teach people about anthrax and how to prevent it.”*

The data consistently highlights the critical role of education and awareness programs, which should be tailored to the specific needs and cultural contexts of communities. They should utilize diverse communication channels to reach a wide audience and promote behavior change that reduces the risk of anthrax transmission.

### 3.8. Conservancy management and community relations

This theme explores the complex relationship between communities and conservancy management, examining the perceived benefits and grievances associated with wildlife conservation, land use, and resource access.

#### 3.8.1. Benefits and grievances.

Participants expressed a range of views on the benefits and grievances associated with wildlife conservation and park management. While some acknowledged the potential economic benefits of tourism and conservation efforts, others voiced concerns about the negative impacts on livelihoods and access to resources.

***Simalaha men*** *: A1: “The park has brought some benefits to our community, such as jobs and tourism revenue. But we also face challenges, such as crop damage by wildlife and restrictions on our access to natural resources”.*

***Mumbwa women*** *: A2: “We used to be able to collect firewood and wild fruits from the park, but now we are not allowed to do so. This makes it difficult for us to meet our basic needs”.*

These contrasting perspectives highlight the need for a more balanced and equitable approach to conservation that considers the needs and concerns of local communities.

#### 3.8.2. Resource access and control.

The data evidently showed community concerns regarding access to and control over natural resources within conservancies. Participants expressed frustration over restrictions on land use, access to water sources, and limited involvement in decision-making processes related to conservation management.

**Simalaha women**: A3: “We need to have a greater say in how the park is managed. We should be involved in making decisions about land use and resource allocation”.

**Monze men**: A4: “The park authorities should consult with us before making any decisions that affect our livelihoods. We need to be partners in conservation, not just beneficiaries”.

Empowering communities to participate in natural resource management and decision-making processes is essential for ensuring the sustainability and legitimacy of conservation efforts. This highlights the need for bottom-up approaches towards building systems of purpose and communities of practice in wildlife conservation [20,30]

#### 3.8.3. Power dynamics.

The data revealed power imbalances between park management, communities, and other stakeholders. Participants perceived a lack of transparency and accountability in conservation management, with limited opportunities for communities to voice their concerns or influence decision-making.

**Simalaha men**: A5: “The park authorities have all the power. They make the rules, and we have to follow them. We need a more equal partnership where our voices are heard”.

**Mumbwa women**: A6: “We need more transparency in how park revenue is used. We want to see the benefits of conservation shared equitably with our community”.

Addressing power imbalances and promoting equitable partnerships between communities and conservation authorities is crucial for building trust and ensuring the long-term success of conservation initiatives and disease control.

### 3.9. Traditional practices and beliefs

This theme explores the role of traditional practices and beliefs in shaping community perceptions and behaviors related to meat consumption, animal health, and disease management.

#### 3.9.1. Meat preparation.

The FGDs discussed traditional methods of preparing and preserving meat, such as drying, smoking, and fermenting. Participants highlighted the cultural significance of these practices and their role in preserving food for times of scarcity.

***Mumbwa men*** *: A1: “We have always dried meat to preserve it for the dry season. This is how our ancestors did it, and it is still an important part of our culture”.*

***Simalaha women*** *: A2: “We smoke meat to preserve it and to add flavor. It is a tradition that has been passed down through generations”.*

While traditional meat preparation methods can be effective for preservation, it is important to ensure they are safe and do not increase the risk of disease transmission. This may involve promoting hygienic practices and raising awareness about the potential risks associated with certain traditional methods and their role in the transmission of diseases like anthrax.

#### 3.9.2. Rituals and customs.

Cultural rituals and customs associated with meat consumption and animal slaughter were also discussed. These practices often have deep cultural significance and play a role in social cohesion and community identity.

***Monze men*** *: A3: “When we slaughter an animal, we share the meat with our neighbors and relatives. This is a way of showing respect and maintaining good relationships”.*

***Simalaha women*** *: A4: “We have certain rituals that we perform when an animal dies. This is to show respect for the animal and to appease the spirits”.*

Understanding and respecting cultural rituals and customs is essential for engaging communities in anthrax prevention and control efforts. Interventions should be designed to be sensitive to local traditions and beliefs, as supported by Sitali [11].

#### 3.9.3. Traditional medicine.

The FGDs acknowledged the role of traditional healers and remedies in addressing animal and human health issues. Participants expressed a belief in the efficacy of certain traditional medicines for treating various ailments, including those related to human anthrax.

***Mumbwa men*** *: A5: “We have traditional healers who can treat anthrax. They use herbs and other natural remedies that have been passed down through generations”.*

***Monze women*** *: A6: “When someone gets sick, we first try to treat them with traditional medicine. If that does not work, then we go to the clinic”.*

Traditional medicine plays an important role in community health practices. It is important to acknowledge and respect this knowledge system while promoting collaboration between traditional healers and modern healthcare providers to ensure the safe and effective treatment of anthrax using a multidisciplinary, one-health approach.

## Conclusions and recommendations

This study highlights the complex socio-ecological dynamics driving anthrax transmission in Game Management Areas in Zambia. Community knowledge about anthrax varies, with widespread misconceptions and cultural beliefs influencing risk perceptions and meat consumption practices. The illegal game meat trade and close human-wildlife-livestock interactions exacerbate disease spread, while economic pressures drive risky behaviors. The study underscores the socioeconomic burden of anthrax, including livestock losses, food insecurity, and strained public health and veterinary responses. Additionally, the findings emphasize the need for targeted education, improved surveillance, and One Health interventions. Addressing anthrax risks requires a multi-sectoral strategy that integrates community engagement, policy enforcement, and sustainable livelihoods to reduce reliance on illegal meat markets and enhance public health resilience.
